# The health-related quality of life burden of co-morbid cardiovascular disease and major depressive disorder in Australia: findings from a population-based, cross-sectional study

**DOI:** 10.1007/s11136-012-0128-4

**Published:** 2012-02-10

**Authors:** Adrienne O’Neil, Christopher E. Stevenson, Emily D. Williams, Duncan Mortimer, Brian Oldenburg, Kristy Sanderson

**Affiliations:** 1 Department of Epidemiology and Preventive Medicine, School of Public Health and Preventive Medicine, Monash University, 99 Commercial Road, Melbourne, VIC Australia; 2 Centre for Health Economics, Faculty of Business and Economics, Monash University, Wellington Road, Clayton, VIC Australia; 3 Menzies Research Institute Tasmania, University of Tasmania, Private Bag 23, Hobart, TAS Australia

**Keywords:** Health-related quality of life, Depression, Cardiovascular disease, Dose–response, Synergistic

## Abstract

**Purpose:**

Health-related quality of life (HRQOL) can be significantly impaired by the presence of chronic conditions such as cardiovascular disease (CVD) and major depressive disorder (MDD). The aim of this paper was to (1) identify differences in HRQOL between individuals with CVD, MDD, or both, compared to a healthy reference group, (2) establish whether the influence of co-morbid MDD and CVD on HRQOL is additive or synergistic and (3) determine the way in which depression severity interacts with CVD to influence overall HRQOL.

**Methods:**

Population-based data from the 2007 Australian National Survey of Mental Health and Well-being (NSMHWB) (*n* = 8841) were used to compare HRQOL of individuals with MDD and CVD, MDD but not CVD, CVD but not MDD, with a healthy reference group. HRQOL was measured using the Assessment of Quality of Life (AQOL). MDD was identified using the Composite International Diagnostic Interview (CIDI 3.0).

**Results:**

Of all four groups, individuals with co-morbid CVD and depression reported the greatest deficits in AQOL utility scores (Coef: −0.32, 95% CI: −0.40, −0.23), after adjusting for covariates. Those with MDD only (Coef: −0.27, 95% CI: −0.30, −0.24) and CVD only (Coef: −0.08, 95% CI: −0.11, −0.05) also reported reduced AQOL utility scores. Second, the influence of MDD and CVD on HRQOL was shown to be additive, rather than synergistic. Third, a significant dose–response relationship was observed between depression severity and HRQOL. However, CVD and depression severity appeared to act independently of each other in impacting HRQOL.

**Conclusions:**

HRQOL is greatly impaired in individuals with co-morbid MDD and CVD; these conditions appear to influence HRQOL in an additive fashion. HRQOL alters with depression severity, therefore treating depression and improving HRQOL is of clinical importance.

## Introduction

Globally, cardiovascular disease (CVD) is the leading cause of premature death [1] and major depressive disorder (MDD) the top-ranking cause of disability [2]. While individually, the health and economic burden of these conditions is great, it is substantially more pronounced when the two conditions co-occur. For example, patients with CVD such as myocardial infarction (MI) who report MDD are significantly more likely to experience poorer health outcomes including increased morbidity, mortality (including suicide) [3] and Coronary Heart Disease (CHD) risk factor profiles, compared with those without depressive symptoms.

### Relationship between CVD, depression and health-related quality of life (HRQOL)

While the relationship between MDD and CVD has been extensively researched over the past 20 years, more recently the role of HRQOL in this relationship has become of interest. Although there are a range of definitions, HRQOL most often comprises key aspects of functioning, including mental, physical and social functioning. For coronary patients, HRQOL outcomes have been shown to be as important as any potential survival outcomes, in some cases, of greater importance. Of survival gain, Rumsfeld and Ho [4] argue that the benefits are “…limited to specific patient subsets and many patients express a desire for quality of life equal to or greater than their desire for quantity of life”. There is compelling evidence that depression is the best predictor of HRQOL in MI populations, both in the short [5] and long term [6]. The importance of the role of depression in the HRQOL of CVD patients has been highlighted when the influence of mental health, as distinct from physical health [7], has been examined. Findings from the Heart and Soul study identified depression, over physiological factors like left ventricular ejection fraction and ischaemia, as having the most important influence on HRQOL of cardiac patients. In fact, this association is such that a dose–response relationship exists between HRQOL and depression.

### Dose–response relationship between HRQOL and depression

Cross-sectional [7] and other studies [8] indicate that depression severity increases synchronously with HRQOL impairments. This dose–response relationship has been demonstrated in cardiac, as well as other, populations. However, the way in which the relationship between depression and HRQOL is attenuated by the presence of CVD remains less clear. Because HRQOL encompasses both physical and mental health functioning, it would be expected that the presence of co-morbid depression and CVD, compared with the presence of major depression alone, would exacerbate the effect between HRQOL and depression observed in previous studies. However, to our knowledge there is limited evidence to support this assertion. An understanding of the way in which depression severity interacts with CVD to influence overall HRQOL is required to aid our knowledge of the complex relationship between CVD, depression and HRQOL.

### The impact of co-morbid depression and CVD on HRQOL: synergistic or additive?

Indeed, the relative impact of chronic medical co-morbidities on HRQOL has been investigated in order to determine whether the impact of disease on HRQOL is synergistic or additive in nature. An additive effect suggests that the combined effect of MDD and CVD on HRQOL would approximate the sum of the independent effect of each of these conditions, whereas a synergistic relationship suggests that the combined effect is “greater than the sum of the independent effect of each of these conditions” [9].

To date, research exploring the additive and synergistic effects of medical co-morbidities on HRQOL has revealed disparate results, across disease populations. For individuals with diabetes and other chronic medical co-morbidities, the impact of co-morbid conditions on HRQOL has been shown to be additive, rather than synergistic [9]. In contrast, research comprising Hepatitis C [10] populations has revealed a synergistic influence of MDD and disease on HRQOL. In those with CVD and diabetes specifically, these conditions have been found to significantly interact with one another to result in poorer functioning [11].

Previous research conducted in the 1980s suggested that MDD and CVD may have an additive effect on well-being and functioning; the combination of heart disease and depression was shown to cause almost twice the social impairment caused by either condition alone [12]. However, the current understanding of the impact of MDD and CVD on HRQOL using appropriate instruments, specifically designed to detect differences in HRQOL, is limited. Identifying whether a synergistic relationship exists between these two conditions in relation to HRQOL is important for two key reasons. A condition such as MDD may affect a patient’s behaviour in an adverse manner, thereby impacting negatively on treatment outcomes for CVD [13]. Alternatively, treating one condition may subsequently impact on the other pre-existing condition, resulting in lower HRQOL than would be expected as a result of the pre-existing condition on its own.

The aim of the paper was to address the current research gaps by using the Assessment of Quality of Life (AQOL) instrument to (1) identify differences in HRQOL between individuals with CVD, MDD, or both, compared to a healthy reference group, (2) establish whether the influence of co-morbid MDD and CVD on HRQOL is additive or synergistic and (3) determine the way in which depression severity interacts with CVD to influence overall HRQOL.

## Methods

### Study design and sampling

Cross-sectional, population-based data from the 2007 Australian National Survey of Mental Health and Well-being (NSMHWB) were used. This methodology has been described in detail elsewhere [14]. Briefly, the sample was based on a stratified, multistage probability sample of persons aged between 16 and 85 living in private dwellings in Australia, excluding very remote areas. The overall response rate was 60%, totalling 8841 participants. AQOL utility scores were available for 8,820 participants.

### Participants

Respondents with depression in the last 12 months were identified using the Composite International Diagnostic Interview (CIDI 3.0) [15], one of the most widely used, structured diagnostic interviews for psychiatric disorders in the world. Diagnostically, MDD is characterised by the presence of severely depressed mood persisting for at least 2 weeks [16]. Respondents were identified as having CVD on the basis of their response to the question ‘have you had or been treated for a (new or recurrent) CVD condition (e.g. heart attack, angina, high blood pressure) over the past 12 months?’ Research has shown a good correlation between self-reported chronic diseases, such as diabetes, heart disease and asthma and those identified in medical records (e.g. κ = 0.85 for diabetes mellitus [17]). This process allowed us to classify people as those (1) without MDD or CVD, (2) with MDD but not CVD, (3) with CVD but not MDD and (4) with both MDD and CVD. The time frame of 12 months was selected for each condition to best reflect participants’ current disease status.

### Data collection instruments

#### Depression and CVD

Between August and December 2007, specially trained ABS interviewers carried out the assessments at participants’ private dwellings. All interviews were conducted using a computer-assisted interview, which involved the use of a notebook computer to record, store and transmit the collected data. The CIDI 3.0 was administered to diagnose depression. Information was collected to differentiate between three types of depressive episodes, based on the number of symptoms experienced by the participant: Severe Depressive Episode (depressed mood; loss of interest in activities; lack of energy or increased fatigue; and additional symptoms (to total at least eight symptoms)); Moderate Depressive Episode (at least two of the first three symptoms given above and additional symptoms (to total at least six symptoms)); Mild Depressive Episode (at least two of the first three symptoms from the above list and additional symptoms (to total at least four symptoms)[14].

#### Health-related quality of life

The Assessment of Quality of Life (AQOL-4D) instrument [18] was used to assess HRQOL. It was originally developed to increase the sensitivity of multiattribute utility measurement and has the ability to detect nuanced differences in HRQOL---including mental health [19]. The AQOL-4D was the first HRQOL instrument to independently model all the sub-dimensions of health (independent living, social relationships, physical senses, psychological well-being, and illness) and combine sub-models to obtain a multiattribute utility score [19]. Scores from the first 4 dimensions form the multiattribute utility score. Each individual dimension is weighted to produce a dimension score between ‘dimension worst’ (0.0) and ‘dimension best’ (1.0) health states. Dimension scores are then combined to obtain an overall utility score ranging from worst possible HRQOL state (−0.04) to death (0.00) to full HRQOL (1.00). The AQOL-4D measure has maintained structural independence between health dimensions while simultaneously obtaining a high degree of descriptive sensitivity [19]. Based on receiver operator characteristic (ROC) curve analyses and relative efficiency estimates, Osborne [20] concluded that this is a sensitive and responsive HRQOL measure. Because of its robust psychometric properties and the brevity of the scale, the AQOL-4D is considered a suitable instrument for epidemiologic studies where HRQOL and utility data are required. The AQOL-4D has also been used in mental health [21] and cardiac populations [22].

#### Co-variates

Demographic information included age, sex, registered marital status, area socioeconomic disadvantage (Decile 1–10; where 1 = most disadvantage and 10 = least disadvantage)) [14], country of birth, main language spoken at home (English, other), rurality (residing in major urban, other urban, other) [14], education (dichotomised into pre- and post-graduate attainment) [14]. Data were also collected to measure participants’ body mass index (BMI) (calculated using the standard equation of weight divided by height squared [23]), psychological distress (Kessler-10)) [24] and current smoking status [14]. Social support was measured according to frequency of social networking with friends and family (nearly every day, 3–4 days a week, 1–2 days a week, 1–3 days a month, less than once a month; or never). Physical activity in the past week (number of times spent walking for recreation, exercise or gain) was measured using a widely used and validated instrument [14, 25].

### Data analysis

Data were provided by the Australian Bureau of Statistics from a Confidentialised Unit Record File. Estimates and standard errors (SE) were derived using a complex estimation procedure to account for the stratified multistage survey design, oversampling and non-response [14], using the Jackknife delete-2 technique. The use of Jackknife techniques is commonly used for the analysis of complex survey data. It involves deleting one sample primary sampling unit (PSU) at a time to form replicates, and reweighting every replicate as necessary in order to make inference to the population represented by the full sample. Using these replicates, it is possible to calculate the standard error, using the delete-a-group Jackknife standard error estimator [26]. Probability (sampling) weights were applied to weight the sample back to the population from which the sample was drawn.

Using methods described by Hosmer and Lemeshow [27], linear regression was performed to assess differences in AQOL utility scores across disease groups, the synergistic effect of disease on HRQOL, and dose–response effects between AQOL utility scores and recent depression severity over the past 12 months. Algorithms for AQOL scoring were obtained from http://www.aqol.com.au/scoring-algorithms.html. Where negatively skewed (AQOL utility scores), data were transformed using the appropriate log transformations (log_e_ transformation^3). Post-estimation tests were conducted for final regression models. Measures of magnitude were presented as adjusted Coefficients with Jackknife SEs and 95% confidence intervals (CIs). Synergistic effects of CVD and MDD were assessed by the addition of a CVD/MDD interaction with a model containing separate main effects terms: CVD over the past 12 months (yes/no) and MDD over the past 12 months (yes/no). Stata 11 (survey procedures) was used for all statistical analyses. STROBE guidelines (20) were applied for the reporting of cross-sectional studies.

## Results

The key characteristics for all survey participants are displayed in Table 1, by disease status (*n* = 8841). Those belonging to the healthy reference group comprised the greatest proportion of individuals with a post-graduate education and non-English speaking individuals. Those with co-morbid CVD and MDD had the highest proportion of individuals belonging to a lower socio-economic bracket, reporting lowest physical activity frequency, and highest psychological distress and BMI. Those with MDD only reported the youngest mean age. Of those belonging to this sub-group, almost two-thirds were single. This sub-group comprised the greatest proportion of smokers and the most frequent physical activity over the previous week. Those with CVD only were least likely to be single, reported the highest mean age of CVD onset and mean age. Approximately 10% of the overall sample (10.3%, 95% CI: 9.6, 11.1) reported having ever received helpful or effective treatment for depressive symptoms (sadness/lack of interest); 28.3% (95% CI: 20.4, 36.2) of the MDD and CVD group and 30.7% (95% CI 27.6, 33.9) of the MDD only group.

**Table 1:** Key characteristics of all survey participants, by disease group (*n* = 8841)

	(1) Neither MDD nor CVD *n* = 6,079 Mean/percentage (95% CI)	(2) MDD only *n* = 1326 Mean/percentage (95% CI)	(3) CVD only *n* = 1,223 Mean/percentage (95% CI)	(4) Co-morbid MDD and CVD *n* = 213 Mean/percentage (95% CI)
Age	42.50 (42.17, 42.81)	36.68 (35.71, 37.64)	62.06 (60.86, 63.25)	54.63 (52.08, 57.18)
Sex (male)	50.62% (49.70, 51.56)	45.44% (41.44, 49.43)	51.00 (47.46, 54.44)	40.89 (30.32, 51.46)
AQOL utility score^a^	0.87 (0.87, 0.88)^c^	0.69 (0.67, 0.71)	0.78 (0.76, 0.80)	0.57 (0.51, 0.64)
Country of birth				
Australia	71.24 (69.31, 73.16)	80.86 (77.60, 84.12)	71.64 (66.62, 76.65)	75.00 (66.09, 83.90)
Other English speaking country	11.20 (10.15, 12.26)	9.74 (7.71, 11.76)	13.52 (10.84, 16.19)	13.25 (6.67, 19.83)
Non-English speaking country	17.56 (15.83, 19.28)	9.40 (7.17, 11.64)	14.84 (10.64, 19.04)	11.75 (6.24, 17.26)
Main language spoken at home				
English	90.40 (89.15, 91.66)	94.17 (91.62, 96.73)	92.69 (89.45, 95.94)	96.37 (92.54, 100)
Registered marital status (single)	46.51 (44.97, 48.05)	64.71 (60.15, 69.26)	28.73 (25.00, 32.46)	41.16 (31.63, 50.69)
Post-graduate qualifications (yes)	57.03 (55.64, 58.43)	51.94 (48.28, 55.60)	46.82 (42.50, 51.14)	48.44 (37.75, 59.13)
Level of area social economic disadvantage (Decile 1–5)^b^	44.68 (42.48, 46.88)	46.74 (42.31, 51.18)	49.15 (44.30, 54.00)	57.48 (46.35, 68.60)
Psychological distress (moderate to high distress)	20.67 (19.23, 22.11)	64.31 (60.74, 67.87)	24.08 (19.81, 28.35)	68.95 (56.89, 81.20)
Smoke (yes)	20.59 (19.02, 22.17)	38.46 (34.36, 42.56)	10.60 (8.15, 13.04)	28.00 (16.07, 39.93)
Body mass index^a^	26.02 (25.83, 26.21)	25.88 (25.46, 26.30)	28.60 (28.14, 29.06)	30.37 (28.38, 32.36)
Age of first CVD event	–	–	50.81 (49.54, 52.09)	45.71 (43.22, 48.20)
Frequency of physical activity in past week^a^	5.21 (4.87, 5.54)	5.56 (4.95, 6.18)	4.27 (3.59, 4.94)	4.17 (2.66, 5.68)
Depression severity (*n* %)				
None	6,079 (100%)	–	1,223 (100%)	
Mild	–	465 (35%)	–	69 (33%)
Moderate	–	473 (36%)	–	88 (41%)
Severe	–	388 (29%)	–	56 (26%)

Person weighted, survey corrected means and 95% confidence intervals

^a^Does not include all participants due to missing data

^b^Most disadvantaged

^c^The population norm for the AQOL utility score is 0.83 (SD 0.20) [33]

AQOL utility scores were available for 8,820 participants. Of those respondents for whom AQOL utility scores were not available (*n* = 21), none belonged to the co-morbid CVD/MDD group; 13 belonged to the reference group, three were from the MDD only group and five were from the CVD only group. Overall, they were older, comprised more men, belonged to a lower socio-economic bracket and reported higher psychological distress, when compared with those for whom full data were available. After controlling for sex, age, marital status, education, area disadvantage, rurality, smoking, social support, BMI, employment status, a multivariate linear regression model revealed significant impairments in AQOL utility scores in all three disease groups, compared with a healthy reference group (Table 2). Of all the groups, individuals with co-morbid depression and CVD reported the lowest AQOL utility scores (Coef: −0.32, 95% CI: −0.40, −0.23). Those with MDD only (Coef: −0.27, 95% CI: −0.30, −0.24) and CVD only (Coef: −0.08, 95% CI: −0.11, −0.05) also reported reduced AQOL utility scores. In addition to exploring the impact of disease on overall AQOL utility score, we re-ran these analyses for each AQOL dimension (independent living, social relationships, physical senses, mental health). We observed similar trends in impairment by disease group for each dimension of HRQOL (data not shown).

**Table 2:** Linear regression model for the relationship between log-transformed AQOL utility scores and disease status (*n* = 8820)

Disease group	Univariate coefficient	Adjusted coefficient^a^	*p*	95% CI^a^	
Neither CVD nor MDD	1.0	1.0			
MDD only	−0.27	−0.27	<0.00	−0.30	−0.24
CVD only	−0.15	−0.08	<0.00	−0.11	−0.05
Co-morbid MDD and CVD	−0.40	−0.32	<0.00	−0.40	−0.23

^a^Adjusted for sex, age, marital status, education, area disadvantage, rurality, smoking, social support, BMI, employment status

To explore whether the impact of this co-morbidity was additive or synergistic, we undertook another multivariate linear regression analysis. After adjusting for sex, age, marital status, education, area disadvantage, rurality, smoking, social support, BMI and employment status, the model revealed a non-significant interaction between CVD and MDD (adjusted coefficient: 0.03, 95% CI: −0.06, 0.12; *p* = 0.52), suggesting the relationship may be additive, rather than synergistic.

**Fig. 1:**
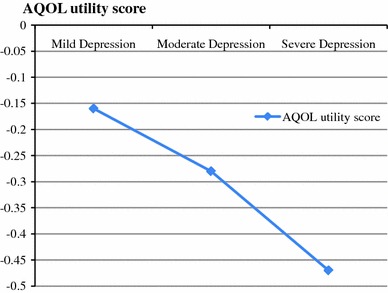
Dose-reponse relationship between AQOL utility score and depression severity

Next, we explored whether a dose–response relationship exists between MDD severity and HRQOL. After adjusting for sex, age, marital status, smoking, social support, BMI, employment status and CVD-MDD severity interaction, a regression model revealed a significant relationship between MDD severity and HRQOL (Mild; Coef: −0.16, 95% CI: −0.20, −0.12, Moderate; Coef: −0.28, 95% CI: −0.32, −0.24, Severe; Coef: −0.47, 95% CI: −0.51, −0.43) (Table 3). This relationship is displayed in Fig. 1, where an increase in depression severity is shown to be associated with greater deficits in AQOL utility score. We then entered CVD and MDD severity into the model as an interaction term. The interaction failed to reach significance, suggesting that CVD and depression severity may act independently to impact HRQOL, rather than synergistically.

**Table 3:** Linear regression model assessing dose–response relationship between depression severity and AQOL utility scores (*n* = 8820)

Depression severity	Univariate coefficient	Adjusted coefficient^a^	*p*	95% CI	
Mild	−0.14	−0.16	<0.00	−0.20	−0.12
Moderate	−0.29	−0.28	<0.00	−0.32	−0.24
Severe	−0.50	−0.47	<0.00	−0.51	−0.43
CVD-Mild depression interaction	0.05	0.06	0.47	−0.11	0.23
CVD-Moderate depression interaction	−0.03	−0.01	0.73	−0.09	0.07
CVD-Severe depression interaction	0.05	0.04	0.26	−0.03	0.12

^a^Adjusted for sex, age, marital status, smoking, social support, BMI, employment status, CVD-MDD severity interaction

## Discussion

Our finding that impairments in HRQOL are greatest for those with co-morbid MDD and CVD is consistent with studies of clinical populations, which have demonstrated the magnified effects of this co-morbidity on HRQOL [5]. These results add to the literature by providing robust evidence of these disease-related impairments at the population level. Our findings are potentially more representative than other studies, where estimates derived from clinical populations may be skewed towards more severe health states. While our second finding that the influence of MDD and CVD on HRQOL is additive, rather than synergistic, is consistent with some studies of other medical co-morbidities on HRQOL [9], to our knowledge, ours is the first to attempt to disentangle the independent effects of CVD and MDD on HRQOL, using a measure of HRQOL which specifically detects nuanced differences in HRQOL [19]. Third, our finding of a significant dose–response relationship between depression severity and HRQOL, while consistent with studies comprising Coronary Artery Disease populations [7], adds to the existing literature because, to our knowledge, this is also the first time this has been demonstrated in those with CVD, at the population level. We chose to compare our findings with those of clinical populations as we would expect those studies comprising MI patient samples to show a more pronounced dose–response effect between depression and HRQOL because of the recency of the cardiac event and the likelihood of depression occurring in the first six months, post-MI [28]. Our study provides a broader view of the relationship between depression and HRQOL in a wide sample of individuals affected by a range of CVD, not restricted only to those hospitalised as a result of MI. Thus, our results are potentially more generalisable to the wider CVD population, in addition to those experiencing post-MI depression.

Indeed, our findings highlight the influence of co-morbid depression (particularly of increasing severity) and CVD on HRQOL status. There are a range of explanations regarding the mechanisms that link MDD and CVD to HRQOL. The observed deficits in HRQOL for those with co-morbid CVD and MDD may reflect physical illness of greater severity, which subsequently intensifies depression severity. Conversely, increasing depression severity may exacerbate an individuals’ perception of their functional impairments. Indeed, our findings suggest that depression severity is a stronger contributor to HRQOL impairments than CVD; the presence of CVD does not appear to attenuate the dose–response effects previously observed between depression and HRQOL [7]. Depression management has been shown to improve HRQOL in patients with depression [29]. However, in co-morbid populations, given that the effects of MDD and CVD appear to act independently of each other, we recommend that cardiac rehabilitation programmes, which address lifestyle factors be incorporated into depression treatment programmes if overall HRQOL status (particularly physical functioning) is to be improved. We further recommend that randomised controlled trials, which evaluate the benefits of combined depression treatment and lifestyle modification programmes in CVD patients exhibiting depression be undertaken, with the inclusion of HRQOL endpoints. Furthermore, the cost-effectiveness of such a programme should be evaluated from the perspective of each of the responsible fund-holders (e.g. hospital cost-centres, hospital/health network, state/federal health budgets) to determine the possible business case for wide scale implementation [30]. Where routine depression treatment is seldom available after a cardiac event, and participation in cardiac rehabilitation programmes is often low, offering alternative approaches to treatment after a cardiac event could potentially reduce the HRQOL burden. Contemporary approaches to treatment using tele-health or web-based interventions could promote uptake and adherence to rehabilitation, where various logistic and other barriers (including depressive symptoms) have been shown to impede participation [31].

A strength of this study was the administration of a diagnostic interview to assess MDD and the use of the AQOL instrument and the utility scores it generates. A further advantage of this study was its robustness and representativeness due to the use of a large, probability sample from the general population. Third, the use of the AQOL instrument to measure HRQOL is advantageous; it has been shown to have sound instrument sensitivity where other HRQOL instrument sensitivity is questionable [32]. However, several limitations were observed. The cross-sectional design of the study precludes us from determining causality, or the long-term impact of co-morbid depression and CVD on HRQOL in this population. Additional research could use longitudinal or panel studies to explore HRQOL trajectory and associated costs over time. Secondly, in the absence of objective data, CVD status was determined by self-report. Further, CVD was defined as “any heart or circulatory condition”. These measures may have led to recall bias, misclassification or incorrect identification of CVD and possible dilution of the CVD effect.

## Conclusions

Our assessment of the impact of co-morbid MDD and CVD on HRQOL at the population level, as distinct from clinical populations, provides an important snapshot of the current burden of, and interaction between, CVD and a high prevalence mental health disorder like depression in the general population. Due to their increasing prevalence in ageing Western populations, minimising the burden of their consequences at the population level through prevention and appropriate management remains paramount.

## Abbreviations

CVD: Cardiovascular disease
MDD: Major Depressive Disorder
MI: Myocardial Infarction
CHD: Coronary Heart Disease
HRQOL: Health-related quality of life
NSMHWB: National Survey of Mental Health and Well-being
CIDI 3.0: Composite International Diagnostic Interview 3.0
AQOL-4D: Assessment of Quality of Life
BMI: Body Mass Index
SE: Standard Errors
CI: Confidence Intervals
STROBE: Strengthening the Reporting of Observational Studies in Epidemiology
